# Does millet consumption contribute to raising blood hemoglobin levels compared to regular refined staples?: a systematic review and meta-analysis

**DOI:** 10.3389/fnut.2024.1305394

**Published:** 2024-02-12

**Authors:** Seetha Anitha, Takuji W. Tsusaka, D. Ian Givens, Joanna Kane-Potaka, Rosemary Botha, Nur Liana Binti Sulaiman, Shweta Upadhyay, Mani Vetriventhan, Ananthan Rajendran, Devraj J. Parasannanavar, Thingnganing Longvah, Kowsalya Subramaniam, Raj Kumar Bhandari

**Affiliations:** ^1^Asia-Pacific Association of Agricultural Research Institutions (APAARI), Bangkok, Thailand; ^2^International Crops Research Institute for the Semi-Arid Tropics (ICRISAT), Patancheru, India; ^3^Asian Institute of Technology (AIT), Khlong Luang, Thailand; ^4^Institute of Food, Nutrition, and Health, University of Reading, Reading, United Kingdom; ^5^International Rice Research Institute (IRRI), Los Baños, Philippines; ^6^One Acre Fund, Kigali, Rwanda; ^7^National Institute of Nutrition (NIN), Hyderabad, India; ^8^Avinashilingam Institute for Home Science and Higher Education for Women, Coimbatore, Tamil Nadu, India; ^9^National Technical Board of Nutrition, Government of India (GoI), Mumbai, India

**Keywords:** iron deficiency anemia, millets, hemoglobin, dietary iron, difference-in-differences

## Abstract

Millets are recognized for their health and nutritional values, and the United Nations declared 2023 the International Year of Millets. Among the several health and nutritional benefits of millets, their impact on hemoglobin concentration is important since anemia is a major public health issue in many countries. To investigate the effect of millet (including sorghum) consumption on hemoglobin concentration in the blood, a systematic review and meta-analysis were conducted. Thirteen published studies featuring randomized control trials involving 590 individuals in the intervention group and 549 control individuals were eligible for the meta-analysis. The difference-in-differences analysis revealed highly significant (*p* < 0.01) positive effects of millet consumption on hemoglobin concentration, with an effect size of +0.68 standardized mean difference units. The change in hemoglobin concentration observed in the intervention group was +13.6%, which is statistically significant (*p* < 0.0005), compared to that in the control group, which was +4.8% and not statistically significant (*p* = 0.1362). In four studies, the consumption of millets in the intervention group demonstrated a change from mild anemia to normal status among children, whereas there was no change in the control group. The findings provide evidence that the consumption of millets can improve blood hemoglobin concentration, likely resulting from increased iron intake. Further research is needed involving the assessment of iron content and bioavailability to better understand the effect variation among millet types and the mechanisms involved.

## 1 Introduction

Iron deficiency anemia (IDA) is a global public health issue that affects children and young women in particular. According to the World Health Organization (WHO) estimates for 2019, global anemia prevalence in women of reproductive age and children was 29.9 and 39.8%, respectively, accounting for half a billion women and 269 million children (1). A deficit in dietary iron intake remains a significant challenge, exacerbated by the growing global consumption of refined and highly processed foods, leading to micronutrient deficiency in vulnerable populations. Staple cereals continue to dominate food consumption in developing countries; these mainly include refined wheat, rice, and maize, while other nutrient-rich crops such as millets and sorghum are underutilized (2–4) Supplementation with iron, widespread fortification of foods with iron, and dietary diversity are evidence-based approaches to combating anemia. Interventions aimed at enhancing dietary diversity also facilitate the intake of a wide range of micronutrients, rather than focusing on just one. Studies in different parts of the world have shown that enhancing dietary diversity has resulted in improved hemoglobin levels (5, 6). In this context, millets play an important role in promoting dietary diversity, which in turn ensures the consumption of a wide spectrum of essential vitamins, minerals, and other nutrients. Regions in which millets historically constituted a significant part of the diet have seen their prominence in the dietary landscape gradually diminish over time (7). Whole grain millets have advantages over refined cereals, as they have higher levels of nutrients such as iron, zinc, and protein, to name a few (2). The effect of consuming millets that are rich in iron on blood hemoglobin concentration and anemia has been studied by many researchers. However, this is the first time that evidence on the impact of consuming millet on hemoglobin levels compared to the consumption of other common staples has been collated.

A previous systematic review and meta-analysis showed that millets reduce fasting blood glucose levels (8) and hyperlipidemia (9) and improve growth in children (10). In terms of their effects on anemia, Anitha et al. (11) conducted a systematic review of the potential of millets in raising blood hemoglobin levels, showing that the levels increased from the baseline to the endline. However, the study did not account for changes in the control group. The current systematic review and meta-analysis extends the study conducted by Anitha et al. (11) by accounting for the control group.

The review question, therefore, is, “Does the consumption of millets have positive effects on blood hemoglobin concentration in the controlled assessment design as well, where the control group consumes common staples?

## 2 Materials and methods

### 2.1 Study period and protocol

**Figure 1:**
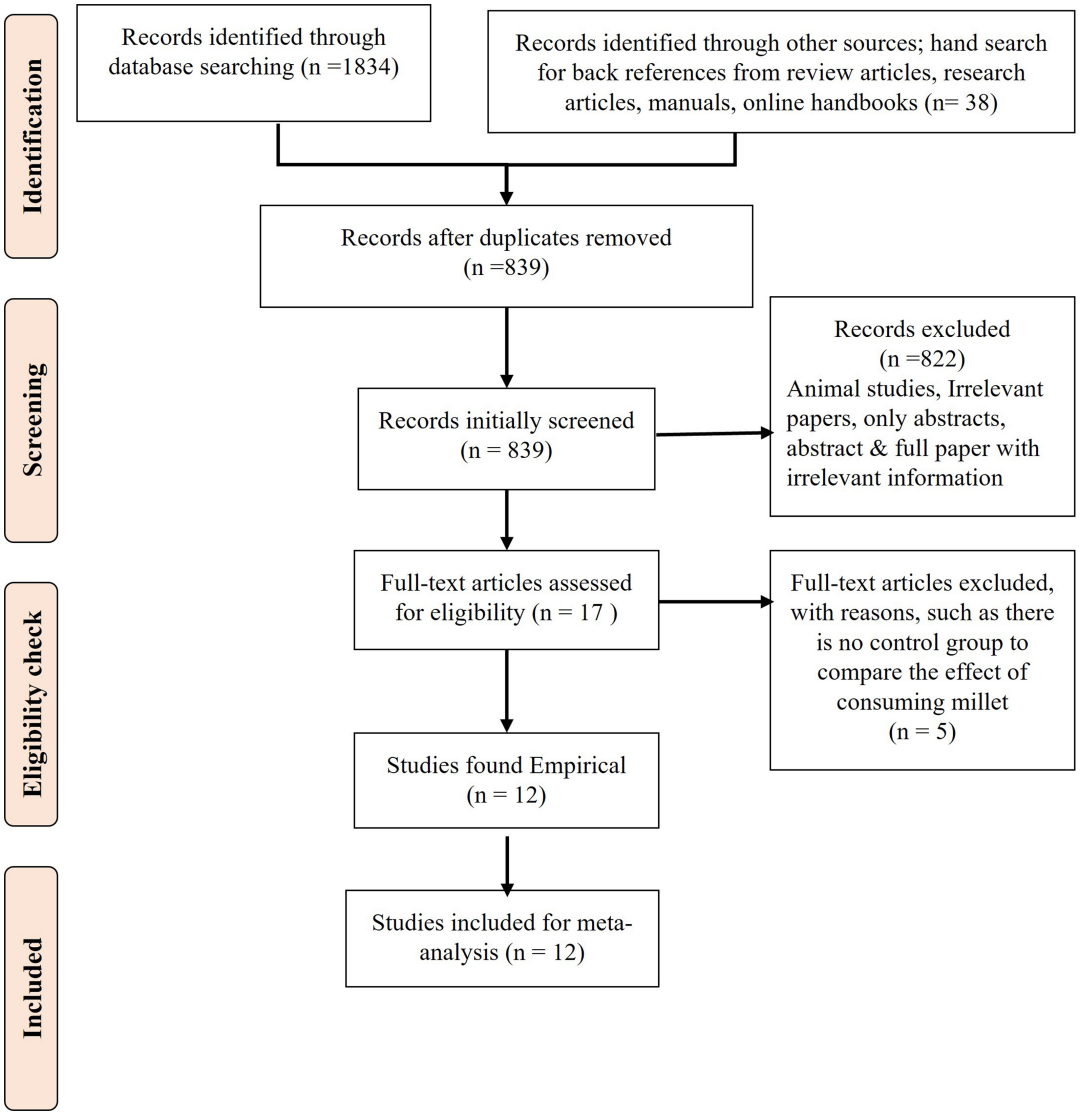
PRISMA flow diagram of the study process.

The systematic review and meta-analysis were conducted from October 2017 to September 2022. The PRISMA checklist (12, 13) was used to write the protocol, which was registered with the unique identification number “reviewregistry1114” in the online platform “research registry.” Figure 1 describes the process of the systematic review.

### 2.2 Search

Studies published in English until September 2022 were obtained through major search engines, namely, Google Scholar, Scopus, Web of Science, PubMed, and CAB abstracts.

### 2.3 Search strategy

The search was conducted using predetermined terminology such as “hemoglobin level AND millets,” “Anemia AND millets,” “millet consumption AND hemoglobin level,” and “millet consumption and anemia.” The search was repeated by replacing millet with the specific type of millet.

### 2.4 Inclusion criteria

The review included randomized controlled trials conducted on the effect of the consumption of millets on blood hemoglobin level, where the control group consumed a regular diet, and studies conducted on any age group (children, adolescents, and adults) or gender of any geographical region. Only human studies were considered as were peer-reviewed journal articles and completed MSc or PhD theses that were available online.

### 2.5 Exclusion criteria

Review articles, animal studies, publications with incomplete data on hemoglobin levels, and papers using only an intervention group were excluded.

### 2.6 Data items

From each eligible study, information on the authors, year of publication, age group and gender of the study participants, country details, sample size in both intervention and control groups, and study methodology were recorded in an Excel sheet (14, 15) along with the mean and standard deviation difference in hemoglobin levels in g/dl.

### 2.7 GRADE assessment

A GRADE assessment was conducted to evaluate the quality of the published articles included in the meta-analysis, based on criteria described by Cochrane (16). Two co-authors of this systematic review independently conducted the GRADE assessment and had no disagreement, obviating the need for another co-author’s input. Quality was assessed based on the ranking of eight criteria, namely, risk of bias, inconsistency, indirectness, imprecision, publication bias, large magnitude of effects, dose–response, and effects of all plausible confounding factors. Ratings were given to downgrade the first five criteria and/or upgrade the sixth to eighth criteria according to the assessment for each criterion. Publication bias was assessed using a funnel plot (17).

### 2.8 Data analysis

A total of 12 published articles with 13 studies were found eligible for inclusion in the meta-analysis. The mean change and standard deviation of hemoglobin levels were observed in the intervention and control groups consuming a normal diet (i.e., refined rice and/or wheat). The effects of the treatment were evaluated based on the difference-in-differences (DID) method to address the bias arising from changes in the control group and any initial difference between the two groups. The statistical significance of the DID was examined by the Wilcoxon matched-pairs signed rank (WSR) test. Furthermore, the treatment effect was formally estimated by the panel DID regression analysis. Standard errors (SEs) were clustered at the individual level to obtain the SE robust to heteroscedasticity. The Hausman specification test was used to choose between the fixed effect and random effect models for the panel regression. The meta-analysis was conducted using R Studio version 4.2.1 (18) to obtain forest plots to determine intervention effects and funnel plots to determine publication bias. The mean change in hemoglobin level (g/dl) before and after the intervention was shown for each group, along with the standard deviation and sample size, to determine the standardized mean difference (SMD) (19–21). The results of the fixed and random effect models were obtained to describe the effect size (22, 23). In addition, the participants were subgrouped into children, adolescents, and adults to determine the intervention effects by age.

Expected outcome: Impact of millet consumption on hemoglobin levels compared to other staple consumption in any geographical location.

## 3 Results

**Figure 2:**
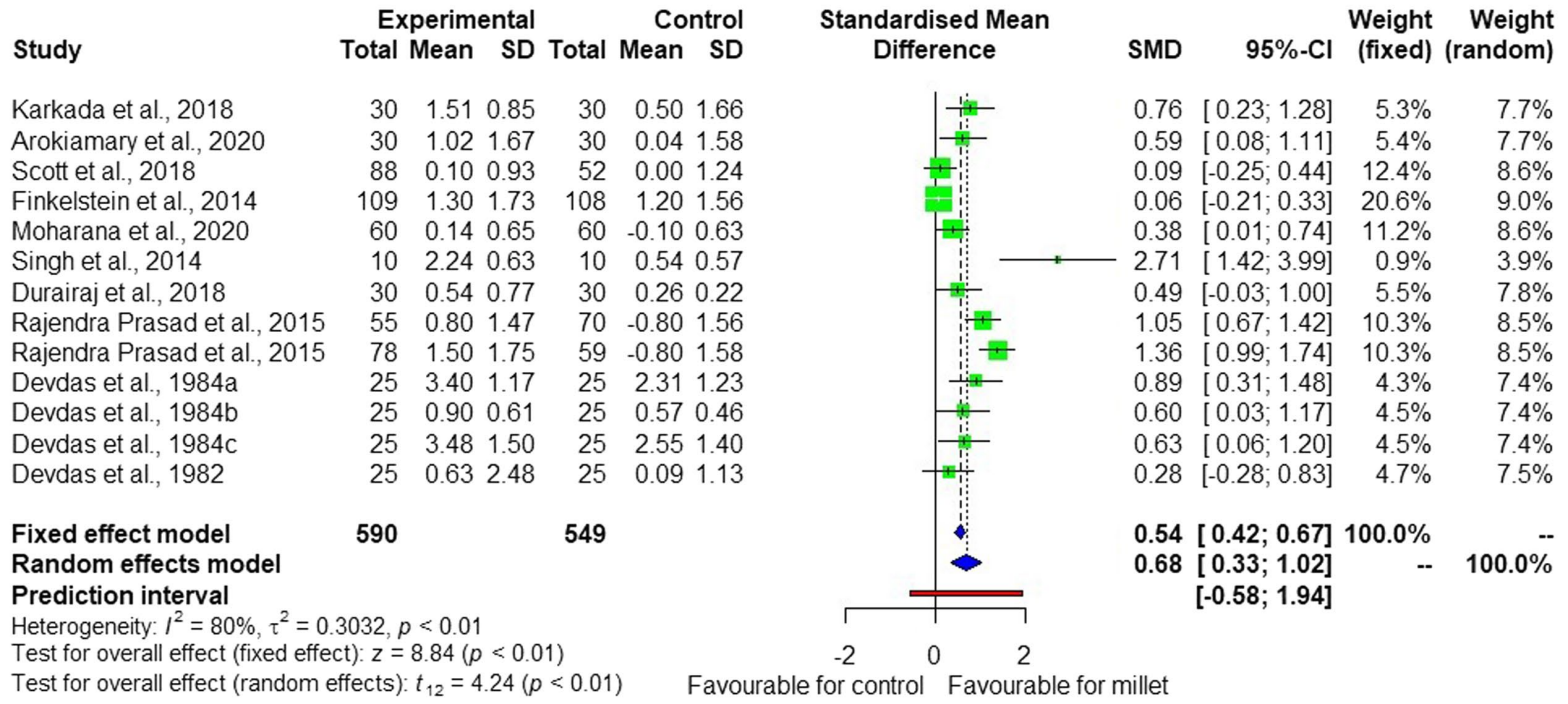
Forest plot showing the effects of millet consumption on blood hemoglobin level.

The meta-analysis of the 13 studies from 12 publications (23–35) (Figure 2) shows high heterogeneity (I^2^ = 80%). The random effect model used to interpret the results showed significant (*p* < 0.01) effects of millet consumption on blood hemoglobin concentration, with an average standard mean difference (SMD) of +0.68 and a confidence interval of [+0.33; +1.02].

Descriptive statistics (Table 1) show that the mean hemoglobin concentration in the intervention group was 9.95 ± 1.07 g/dL before consuming non-refined millet-based food, which increased to 11.31 ± 1.09 g/dL after the intervention. In the control group, it was 10.20 ± 0.99 before the intervention and 10.66 ± 1.09 after the intervention.

**Table 1:** Descriptive statistics for blood hemoglobin concentration (g/dl).

	Treatment group (*n* = 13)		Control group (*n* = 13)	
	Pre-treatment	Post-treatment	Pre-treatment	Post-treatment
Mean	9.95	11.31	10.20	10.66
Standard deviation	1.07	1.26	0.99	1.09

The WSR test shows that the percentage change in the hemoglobin concentration in the treatment group was +13.6% (*p* = 0.000), whereas the change in the control group was not statistically significant (*p* = 0.136) (Table 2). The DID result suggests that the average treatment effect of millet-based diets on blood hemoglobin concentration was +8.8%.

**Table 2:** Changes and difference-in-differences (DID) in blood hemoglobin concentration using the Wilcoxon matched-pairs signed rank (WSR) test.

Treatment group (*n* = 13)		Control group (*n* = 13)		Effect of treatment	
Mean % change	WSR *p*-value	Mean % change	WSR *p*-value	DID	WSR *p*-value
+13.6^***^	0.000	+4.8	0.1362	+8.8	0.017^**^

*** and ** indicate *p* < 0.01 and 0.05, respectively.

Table 3 provides a formal estimation of the treatment effects on blood hemoglobin concentration. The Hausman test was not significant, suggesting the use of the random effect model. The DID estimator indicates that the effect of millet-based diets on hemoglobin concentration was +0.862 g/dL on average.

**Table 3:** Effect of the treatment on hemoglobin concentration using random effect DID regression.

	Coeff.	Robust SE	*p*-value
Treatment (1 if treated, 0 if control)	−0.209	0.668	0.754
Timing (1 for post-treatment, 0 for pre-treatment)	0.489	0.285	0.086
Difference-in-differences (DID)	0.862^**^	0.419	0.040
Constant	10.167	0.495	0.000
Dependent variable =: Blood hemoglobin concentration (g/dl) Hausman test χ^2^ = 0.00 (*p* = 1.000) Number of observations = 52; number of individuals = 26 σ_u_ = 1.49; σ_e_ = 0.75; Wald χ^2^ (d.f. = 3)			

Robust SE = The standard errors robust to individual-level clustering. ** = *p* < 0.05.

**Figure 3:**
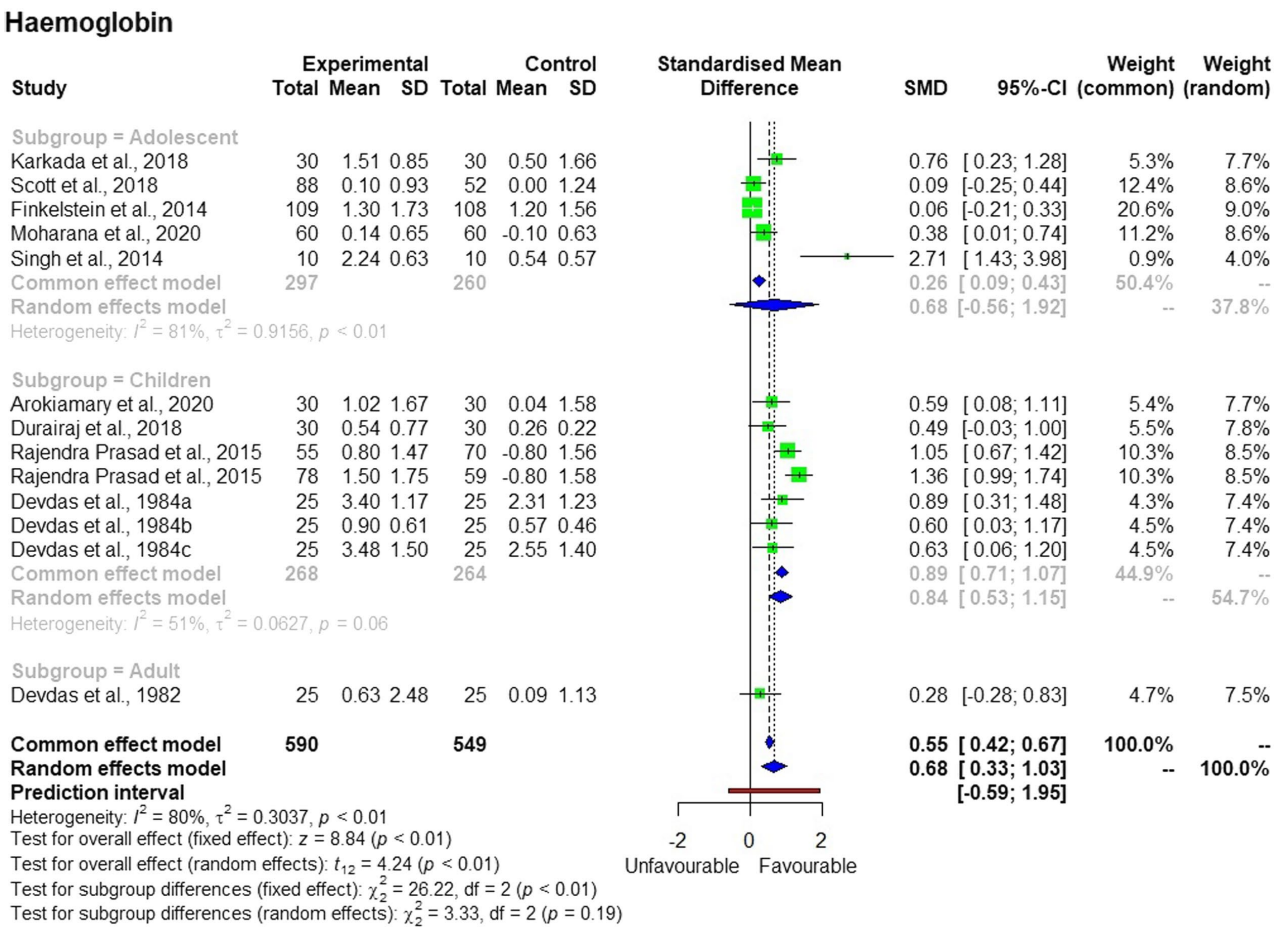
Subgroup analysis of the effects of millet consumption on different age groups.

Interestingly, the subgroup analysis showed that the treatment effects were significant only in the children’s group but not in the adolescent and adult groups (Figure 3). The changes in hemoglobin concentration differed among the three subgroups.

## 4 Discussion

The studies included 297 adolescents, 268 children, and 25 adults in the intervention groups who consumed millet-based food. The control groups had a total of 260 adolescents, 264 children, and 25 adults. Four studies used pearl millet, two used finger millet, one used mixed millet, and one used sorghum (Supplementary Table S1). The millets, specifically finger millet, pearl millet, and sorghum, are generally not refined in India; therefore, the current study assumes that these are whole grain or whole grain flour. However, rice is consumed in refined form in India. One study was conducted for 25 days and another for 45 days, while the rest of the studies were conducted from 100 days to 4.5 years. The mean hemoglobin concentration in the intervention group before the intervention was 9.95 ± 1.07 g/dL, which increased to 11.3 ± 1.09 g/dL, indicating that, overall, individuals with mild anemia saw an improvement to normal levels. In particular, four studies conducted on children reported an increase in hemoglobin levels, demonstrating a shift from mild anemia to a normal status. In the control group, the mean hemoglobin concentration was 10.2 ± 0.99 g/dL before the treatment, which increased to 10.6 ± 1.09 g/dL after the consumption of their regular staple. This also shows that the anemia status in the control group did not change.

The difference between children and adults in the subgroup analysis could be due to the limited number of studies, especially on adults. The low heterogeneity in the children’s group could have arisen from three studies that used the same type of millet, the small sample size, the same geographical location, and the same population.

The interventions in all the studies reviewed in this study were through controlled trials. However, the studies did not provide information on whether they were randomized in terms of selection and assigning samples. Furthermore, the blinding of the intervention was generally not conducted, which is understandable due to the obvious distinction between millet-based meals and common staple foods in terms of texture and appearance. One study noted the attrition of the participants, which was not justified by the study. The heterogeneity of the studies was high (80%), which may be due to the difference in the age group of participants. Only one study was conducted on adults.

**Figure 4:**
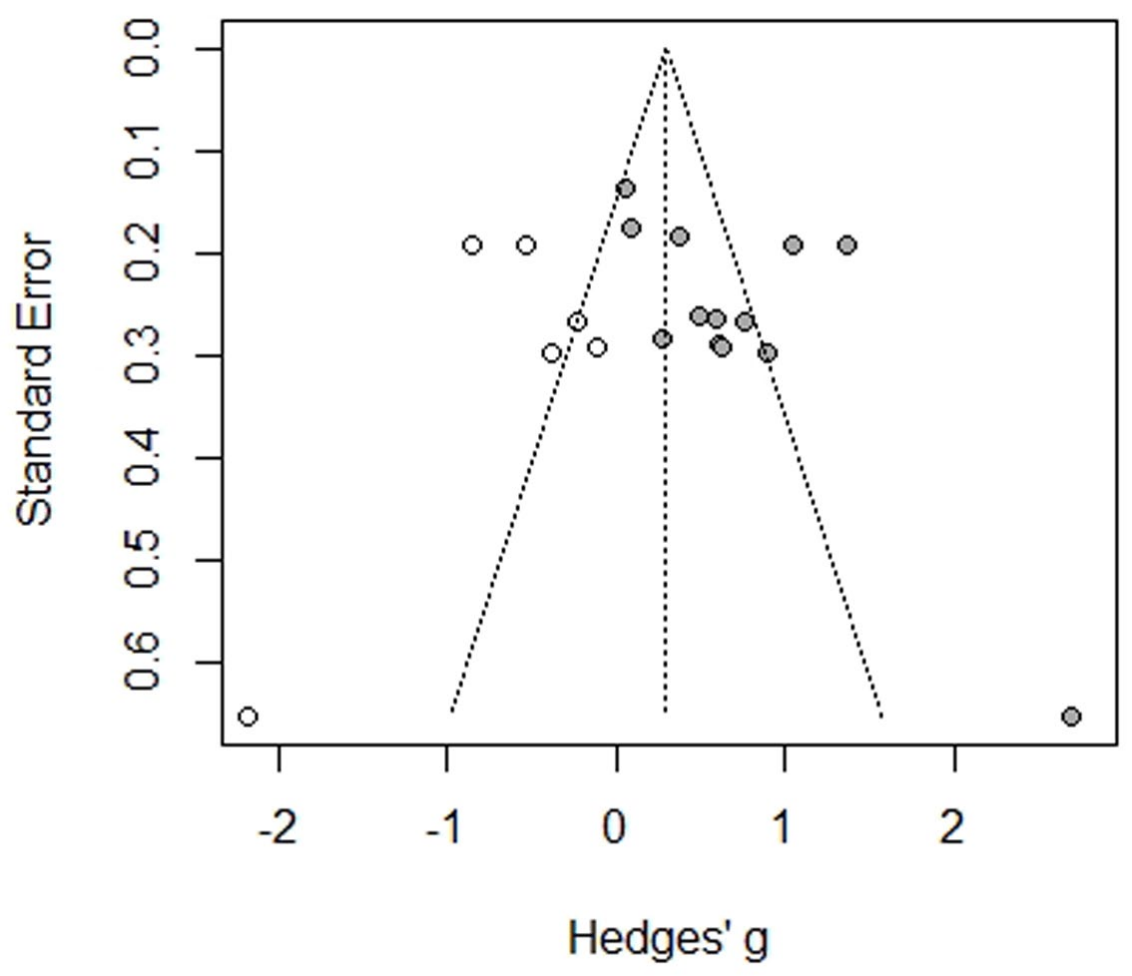
Funnel plot of the studies included in the meta-analysis.

The funnel plot (Figure 4) shows that there is less publication bias, which is evident from the studies that are not scattered in the middle or at the base of the triangle, with the exception of one study. The small sample size in the studies would have made a difference, and therefore, the quality of the obtained evidence was rated as moderate. Although the evidence generated is valuable, it is important to conduct similar studies across various geographical regions with various age groups to strengthen the evidence.

## 5 Conclusion

This study conducted a meta-analysis of 13 studies involving 1,139 participants in the intervention and control groups. Results show that the consumption of millet-based food had a significant positive effect on blood hemoglobin levels. Yet, it is worth noting that these effects were significant only among the children’s group. More robust findings would have been possible if the studies had confirmed the intake of iron in the intervention and control groups.

The limitations of this study include the limited number of studies reviewed in each age group, the limited types of millets tested, and the lack of geographical diversity. Similar studies across diverse geographical regions and sociocultural groups should be conducted to corroborate the evidence of the beneficial effects of millet consumption in addressing anemia.

## Data availability statement

The original contributions presented in the study are included in the article/Supplementary material, further inquiries can be directed to the corresponding author.

## Author contributions

SA: Conceptualization, Data curation, Formal analysis, Validation, Writing – original draft. TT: Formal analysis, Methodology, Writing – review & editing. DG: Writing – review & editing. JK-P: Conceptualization, Funding acquisition, Supervision, Writing – review & editing. RoB: Data curation, Formal analysis, Validation, Writing – review & editing. NS: Methodology, Writing – review & editing. SU: Writing – review & editing. MV: Methodology, Writing – review & editing. AR: Writing – review & editing. DP: Writing – review & editing. TL: Writing – review & editing. KS: Writing – review & editing. RaB: Writing – review & editing.
