# Supplementary material for: Does millet consumption contribute to raising blood hemoglobin levels compared to regular refined staples?: a systematic review and meta-analysis
Source: Front Nutr. 2024 Feb 12;11:1305394. doi: 10.3389/fnut.2024.1305394 (PMC10900984; doi:10.3389/fnut.2024.1305394)
Supplement: Supplementary file 1 [file Table_1.DOCX]

**Table S1: Study participant's characteristics**

| **Author** | **Type of food** | **Age** | **Duration** | **Number of participants** | **Parameters studied** | **Study design** |
| --- | --- | --- | --- | --- | --- | --- |
| Devdas et al.,1982 | Ragi based diet | Pregnant women | 9 months | control = 25, intervention (finger millet) = 25 | Weight, haemoglobin | Feeding intervention to study the efficacy of finger millet-based diet. |
| Devdas et al.,1984a | Ragi based diet | 2 and 1/2 to 7 and 1/2 yrs | 4 and 1/2 years | control = 25, intervention (finger millet) = 25 | height, weight, chest circumference, haemoglobin, serum protein level | Feeding intervention to study the efficacy of finger millet-based diet. |
| Devdas et al.,1984b | Ragi based diet | 6 to 7 and 1/2 yrs | 1 and 1/2 years | control = 25, intervention (finger millet) = 25 | height, weight, chest circumference, haemoglobin, | Feeding intervention to study the efficacy of finger millet-based diet. |
| Devdas et al.,1984c | Ragi based diet | 3 and 1/2 to 6 and 1/2 yrs | 3 years | control = 25, intervention (finger millet) = 25 | weight, height, chest circumference, arm circumference, haemoglobin level | Feeding intervention to study the efficacy of finger millet-based diet. |
| Durairaj et al., 2018 | Mixed millet health mix | < 5 yrs | 6 months | 30 in intervention 30 in control group | Haemoglobin, height and weight | Feeding intervention |
| Karkada et al., 2018 | Finger millet porridge (4 tablespoon or 60g every day) | 13 to 14 yrs | 45 days | 30 in intervention 30 in control group | Haemoglobin | Quasi-experimental design using the two-stage cluster sampling method |
| Moharana, 2020 | Pearl millet ladoo (sweet) | 17 to 19 yrs | 28 days | 60 in intervention and 60 in control | Haemoglobin | Quasi-experimental pretest-post-test design |
| Singh et al., 2014 | Pearl millet ladoo (sweet) | 16 to 19 yrs | 45 days | 10 intervention and 10 control | Haemoglobin | Randomised clinical trial |
| Rajendra Prasad et al., 2015 | Sorghum upma/kchidi, roti | 9 to 12 yrs | 8 months | 80 intervention 80 control | Haemoglobin, serum ferritin, anthropometry | Feeding intervention (there is an attrition rate in the participant's size at the end of the study) |
| Finkelstein et al., 2013 | Pearl millet bhakri (flat bread) | 12 to 16 yrs | 6 months | 109 intervention 108 control | Haemoglobin, serum ferritin | Double-blinded randomised efficacy trial |
| Scott et al., 2018 | Pearl millet bhakri (flat bread) and shev (snack) | 12 to 16 yrs | 6 months | 88 intervention 52 control | Haemoglobin, serum ferritin, transferrin, body iron | Double-blinded randomised intervention |
| Arokiyamari et al., 2020 | pearl millet (100g/day) | 5 to 6 yrs | 100 days | 30 intervention 30 control group | Haemoglobin | Feeding intervention |
